# A Systematic Review of Circulating miRNAs Validated by Multiple Independent Studies in Laryngeal Cancer

**DOI:** 10.3390/diagnostics15030394

**Published:** 2025-02-06

**Authors:** Andreea Banta, Felix Bratosin, Ioana Golu, Ana-Olivia Toma, Eugenia Maria Domuta

**Affiliations:** 1 Doctoral School, Department of General Medicine, “Victor Babes” University of Medicine and Pharmacy Timisoara, 300041 Timisoara, Romania; andreea.banta@umft.ro; 2 Department of Infectious Disease, “Victor Babes” University of Medicine and Pharmacy Timisoara, 300041 Timisoara, Romania; felix.bratosin@umft.ro; 3 Department of Internal Medicine II, “Victor Babes” University of Medicine and Pharmacy Timisoara, Eftimie Murgu Square 2, 300041 Timisoara, Romania; 4 University Clinic of Endocrinology, “Victor Babes” University of Medicine and Pharmacy Timisoara, Eftimie Murgu Square 2, 300041 Timisoara, Romania; 5 Center for Molecular Research in Nephrology and Vascular Disease, “Victor Babes” University of Medicine and Pharmacy, Eftimie Murgu Square 2, 300041 Timisoara, Romania; 6 Discipline of Dermatology, “Victor Babes” University of Medicine and Pharmacy Timisoara, Eftimie Murgu Square 2, 300041 Timisoara, Romania; toma.olivia@umft.ro; 7 Department of Surgery, Faculty of Medicine and Pharmacy, University of Oradea, Piata 1 Decembrie 10, 410073 Oradea, Romania; edomuta@uoradea.ro

**Keywords:** oncology, oncologic surgery, laryngeal cancer, systematic review

## Abstract

**Background and Objectives:** Laryngeal squamous cell carcinoma (LSCC) is a common head and neck cancer with significant morbidity and mortality. Circulating microRNAs (miRNAs) have emerged as promising non-invasive biomarkers for cancer diagnosis and prognosis. This systematic review aims to identify circulating miRNAs associated with LSCC, emphasizing those validated by at least two independent studies to improve reliability and clinical applicability. **Methods:** An extensive literature search was performed in the PubMed, Scopus, and Web of Science databases up to October 2024, using keywords related to LSCC and circulating miRNAs. Studies involving human participants that provided quantitative data on circulating miRNA expression levels and their clinical correlations were included. Data extraction and quality assessment were conducted following standardized protocols, highlighting miRNAs reported in multiple studies. **Results:** Nine high-quality studies encompassing 660 patients with LSCC and 212 controls were included. Several miRNAs were consistently identified across these studies. miR-21-5p was upregulated in four studies and associated with advanced disease stages, lymph node metastasis, and decreased survival rates. miR-125b-5p and miR-126-3p were consistently downregulated, linked to advanced clinical stages and poor tumor differentiation. miR-27a-3p was upregulated in two studies and correlated with poor prognosis, promoting LSCC progression by targeting Smad4. Additionally, miR-33a-5p was identified as a potential diagnostic biomarker with high sensitivity and specificity. These miRNAs show potential as non-invasive biomarkers for the diagnosis and prognosis of LSCC. **Conclusions:** This systematic review highlights specific circulating miRNAs—particularly miR-21-5p, miR-125b-5p, miR-126-3p, miR-27a-3p, and miR-33a-5p—as promising biomarkers for LSCC. The consistent findings across independent studies emphasize their potential clinical utility in early detection, prognostic assessment, and therapeutic targeting. However, further validation in larger and more diverse populations, along with the standardization of detection methods, is necessary before these biomarkers can be implemented in clinical practice.

## 1. Introduction

Laryngeal cancer is a notable public health concern, characterized by its impact on morbidity and mortality worldwide [1,2]. Epidemiological studies have shown that laryngeal cancer primarily affects individuals with long-term tobacco use and alcohol consumption, with additional risk factors including exposure to certain environmental toxins and human papillomavirus (HPV) infection [3,4]. The global incidence of laryngeal cancer varies significantly by geographic region and gender, with higher prevalence observed in men and in regions where tobacco and alcohol use are more prevalent [5,6].

The treatment of laryngeal cancer depends on the stage at diagnosis and includes surgery, radiation therapy, and chemotherapy, often in combination [7,8]. Early-stage laryngeal cancer may be effectively treated with single-modality therapy such as radiation or surgery alone, which can preserve laryngeal function [8,9,10]. However, advanced cases frequently require multimodal treatment, which can result in significant morbidity and impact patient quality of life [11,12]. Prognostically, the five-year survival rate for early-stage laryngeal cancer is favorable, but outcomes for advanced disease remain poor, highlighting the need for improved methods of early detection and treatment stratification [13,14,15].

At the molecular level, laryngeal cancer involves complex genetic and epigenetic changes [16]. Research has increasingly focused on molecular biomarkers that can aid in the early detection, prognosis, and therapeutic targeting of laryngeal cancer. Techniques such as genomic sequencing and gene expression profiling have started to uncover the molecular landscape of laryngeal tumors, offering insights into potential molecular targets for therapy and markers for disease progression [17,18].

MicroRNAs (miRNAs) have emerged as key players in the regulation of gene expression, with their dysregulation implicated in the pathogenesis of various cancers, including laryngeal cancer [19,20]. These small, non-coding RNA molecules can act as oncogenes or tumor suppressors and are notable for their stability in circulating body fluids, which makes them appealing candidates for non-invasive cancer biomarkers. Recent research in oncology has demonstrated the potential of miRNAs to serve as diagnostic, prognostic, and therapeutic biomarkers due to their ability to reflect underlying tumor biology [21,22].

In laryngeal cancer specifically, circulating miRNAs have shown promise as biomarkers that could potentially improve diagnostic accuracy and prognostic assessments. Preliminary studies suggest that specific circulating miRNAs are consistently altered in patients with laryngeal cancer compared to healthy controls, offering a potential tool for non-invasive early detection and the monitoring of disease progression [23]. These findings indicate that miRNAs could also help in predicting treatment response and in stratifying patients based on prognosis.

The primary objective of this systematic review is to critically analyze the existing literature on circulating miRNAs identified by multiple research studies in laryngeal cancer. By consolidating evidence from various studies, this review aims to validate specific circulating miRNAs as reliable biomarkers for laryngeal cancer, thereby assessing their potential roles in diagnosis, prognosis, and therapeutic decision-making.

## 2. Materials and Methods

### 2.1. Eligibility Criteria

This systematic review aimed to identify and analyze all studies investigating circulating microRNAs (miRNAs) associated with laryngeal cancer, specifically laryngeal squamous cell carcinoma (LSCC). We included studies that examined any miRNA species involved in LSCC, focusing on those reported by at least two independent studies to enhance reliability and clinical utility.

The inclusion criteria were as follows—(1) study design: original research articles involving human subjects diagnosed with LSCC, confirmed by histopathological examination; (2) miRNA analysis: studies that provided quantitative data on miRNA expression levels in serum, plasma, extracellular vesicles, or tissues, using methods such as quantitative real-time PCR (qRT-PCR), microarray analysis, or droplet digital PCR; (3) clinical associations: research that explored associations between miRNA expression levels and clinical outcomes, including diagnosis, prognosis, disease stage, survival rates, or response to treatment; and (4) publication language: articles published in English.

The exclusion criteria encompassed the following—(1) non-human studies: studies not involving human participants; (2) publication type: review articles, meta-analyses, conference abstracts, editorials, letters, and case reports; (3) data insufficiency: studies lacking sufficient quantitative data on miRNA expression levels or without clear associations with clinical outcomes; and (4) cancer type: studies involving head and neck cancers other than LSCC, or those that did not provide specific data on laryngeal cancer.

### 2.2. Information Sources

A comprehensive literature search was conducted to identify relevant studies. The electronic databases PubMed, Scopus, and Embase were searched for articles published up to October 2024. Additionally, manual searches of the reference lists of selected articles were performed to identify any studies that might have been missed during the initial database search.

### 2.3. Search Strategy

The search strategy was developed using Medical Subject Headings (MeSH) terms and keywords related to LSCC and miRNAs. No date restrictions were applied. The search was limited to studies published in English involving human subjects. The following terms and their combinations were used—Laryngeal Cancer Terms: “Laryngeal Neoplasms”[MeSH], “Laryngeal Cancer”, “Laryngeal Squamous Cell Carcinoma”, and “LSCC”. miRNA Terms: “MicroRNAs”[Mesh], “MicroRNA”, “miRNA”, “Circulating MicroRNAs”, “Serum MicroRNAs”, “Plasma MicroRNAs”, and “Extracellular Vesicles”. Clinical Outcome Terms: “Biomarkers”[Mesh], “Diagnosis”, “Prognosis”, “Survival”, and “Treatment Outcome”.

Boolean operators (AND, OR) were used to combine search terms effectively. For example, the search string in PubMed included the following: (“Laryngeal Neoplasms”[Mesh] OR “Laryngeal Cancer” OR “Laryngeal Squamous Cell Carcinoma” OR “LSCC”) AND (“MicroRNAs”[Mesh] OR “MicroRNA” OR “miRNA” OR “Circulating MicroRNAs” OR “Serum MicroRNAs” OR “Plasma MicroRNAs” OR “Extracellular Vesicles”) AND (“Biomarkers”[Mesh] OR “Diagnosis” OR “Prognosis” OR “Survival” OR “Treatment Outcome”).

### 2.4. Selection Process

The selection process followed the Preferred Reporting Items for Systematic Reviews and Meta-Analyses (PRISMA) guidelines [24]. Two independent reviewers screened the titles and abstracts of all retrieved articles to assess their eligibility based on the predefined inclusion and exclusion criteria. Full-text articles were obtained for studies that met the inclusion criteria or if eligibility was unclear from the abstract.

During the full-text review, the same two reviewers independently assessed each article for inclusion. Any discrepancies between reviewers were resolved through discussion and consensus. If necessary, a third reviewer was consulted to make a final decision. A PRISMA flow diagram was used to document the number of articles identified, screened, excluded, and included at each stage of the selection process.

### 2.5. Data Collection Process

Data extraction was performed independently by two reviewers using a standardized data extraction form. The extracted data included the following—(1) study characteristics: first author, publication year, country, study design, and quality assessment; (2) patient characteristics: sample size, mean age, gender distribution, disease stage, histological subtype, and sample type; (3) miRNA details: miRNA species studied, detection methods, expression levels, and clinical associations; and (4) key findings: main conclusions of the study.

Disagreements between reviewers during data extraction were resolved through discussion. If consensus could not be reached, a third reviewer was consulted. When necessary, attempts were made to contact the authors of the original studies to obtain missing data or clarify unclear information.

### 2.6. Quality Assessment

The quality of the included studies was evaluated using the Newcastle–Ottawa Scale (NOS) for assessing the quality of nonrandomized studies in meta-analyses [25]. The NOS assesses studies based on three criteria: the selection of study groups, comparability of groups, and ascertainment of exposure or outcome. Each study was awarded a maximum of nine stars. Studies scoring seven or more stars were considered high quality, those scoring four to six stars were considered medium quality, and those scoring less than four stars were considered low quality.

## 3. Results

### 3.1. Study Characteristics

**Figure 1:**
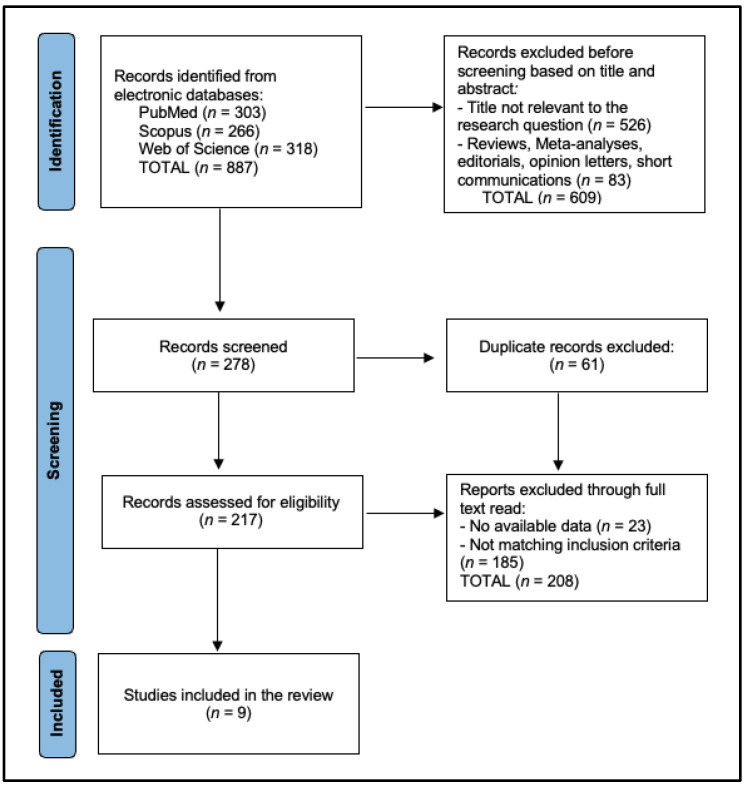
PRISMA flow diagram.

In this systematic review analyzing circulating microRNAs in laryngeal cancer, a total of nine studies were included in the final analysis [26,27,28,29,30,31,32,33,34], as presented in Figure 1. The included studies demonstrated consistently high quality across a diverse array of research designs, predominantly conducted in China [26,27,28,29,31,33,34]. The Newcastle–Ottawa Scale (NOS) was employed for quality assessment, with scores ranging from seven to eight stars, indicating a generally high methodological quality among the included studies. Specifically, three studies, conducted by Gao et al. [26], Wei et al. [28], and Shuang et al. [33], achieved the highest quality score of eight stars, reflecting robust study designs—multicenter observational, prospective, and experimental, respectively.

The prevalence of high-quality ratings across the studies indicates minimal bias, enhancing the credibility of their findings related to circulating miRNAs in laryngeal cancer (Table 1). Notably, the studies spanned from 2013 to 2022, providing a comprehensive temporal perspective that incorporates both early and more recent research insights into miRNA profiles. The geographical concentration in China of the majority of the studies (seven out of nine) could suggest a regional specificity in the research focus or in the genetic and environmental factors associated with laryngeal cancer in the Asian population. Meanwhile, studies from Turkey and Poland—Ayaz et al. [30] and Grzelczyk et al. [32]—each scoring eight stars on the NOS, broaden the cultural and genetic diversity of the data, potentially enriching the applicability of the findings across different populations.

### 3.2. Patient Characteristics

Table 2 illustrates the diversity in sample sizes and demographic profiles, involving a cumulative total of 660 patients with LSCC and 212 controls. For instance, Gao et al. [26] included 236 patients with LSCC with a mean age of 59.4 years and a male-to-female ratio of 223/13, reflecting a male predominance in LSCC cases. This trend is similarly observed in other studies, such as Sun et al. [31], with 38 patients with LSCC, predominantly male (26/12), highlighting common gender distribution patterns in laryngeal cancer studies. The clinical stages varied across the studies, with Gao et al. [26] documenting stages from T-stage I-IV, N0-N2, and M0-M1, providing a broad scope of disease progression within the patient cohort.

Further analysis shows varied approaches to sample types across the studies, which may influence the miRNA profiling outcomes. For example, while Gao et al. [26] and Grzelczyk et al. [32] utilized serum miRNA samples, Wei et al. [28] and Ayaz et al. [30] employed plasma miRNA, potentially affecting the miRNA abundance and detection sensitivities. The histological subtype was consistently LSCC across most studies, except for Zhang et al. [34], who focused on supraglottic carcinoma, a subtype of LSCC, in a comparatively smaller cohort of 10 patients and 10 controls.

### 3.3. miRNA Expression Findings

Regarding miRNA expression findings, Gao et al. [26] identified that elevated levels of miR-21-5p and reduced levels of miR-10a were linked to decreased 5-year survival rates and correlated with TNM cancer staging, suggesting that these miRNAs may serve as prognostic biomarkers for LSCC. Similarly, Wang et al. [27] observed that both miR-21-5p and HOTAIR were upregulated in LSCC, with their higher levels associated with advanced disease stages and lymph node metastasis, reinforcing the potential of these miRNAs in staging and progression assessment. Additionally, the dual detection methods employed, qRT-PCR and ddPCR, ensured robustness in the quantification of miRNA levels, as demonstrated by Wei et al. [28], who reported the significant upregulation of miR-21-5p in both premalignant lesions and LSCC.

Further analysis highlighted the diagnostic potential of miRNAs as non-invasive biomarkers. For example, Grzelczyk et al. [32] utilized both microarray and RT-qPCR techniques to establish a distinct miRNA profile for patients with LSCC, identifying miR-31, LET-7a, and miR-33a-5p as biomarkers with high diagnostic accuracy. On the therapeutic front, Hui et al. [29] found that the lower expression of miR-125b-5p in LSCC tissues was associated with advanced disease stages, poor differentiation, and increased metastasis; moreover, the overexpression of this miRNA inhibited cell proliferation and glycolysis, suggesting a potential therapeutic role (Table 3).

The consolidated findings from multiple studies on microRNAs (miRNAs) implicated in laryngeal squamous cell carcinoma (LSCC) elucidate their pivotal roles in disease progression and diagnostic potential. miR-21-5p, frequently reported by Gao et al. [26], Wang et al. [27], Wei et al. [28], and Zhang et al. [34], consistently appeared upregulated and was strongly associated with advanced disease stages, lymph node metastasis, and reduced survival. This miRNA’s recurrent identification across diverse studies underscores its significance as a prognostic marker, potentially guiding therapeutic decisions and follow-up strategies in clinical settings.

The further analysis of miRNA profiles highlights the utility of miRNAs as biomarkers for LSCC. For instance, miR-223-3p, reported in the studies by Ayaz et al. [30] and Grzelczyk et al. [32], was consistently downregulated and recognized for its diagnostic potential. Similarly, miR-33a-5p, noted in the studies by Grzelczyk et al. [32] and Zhang et al. [34], was upregulated and identified as a potential diagnostic biomarker with high sensitivity and specificity. These findings suggest that certain miRNAs not only contribute to the understanding of the molecular pathogenesis of LSCC but also hold promise for enhancing early diagnosis, thus potentially improving patient outcomes through timely and targeted interventions (Table 4).

## 4. Discussion

### 4.1. Summary of Evidence

This systematic review consolidates evidence regarding miRNAs associated with LSCC. The consistent upregulation of miR-21-5p across multiple independent studies (Gao et al. [26], Wang et al. [27], Wei et al. [28], and Zhang et al. [34]) highlights its potential as a robust biomarker for LSCC diagnosis and prognosis. Elevated miR-21-5p levels were associated with advanced disease stages, lymph node metastasis, and reduced survival rates, suggesting its involvement in tumor progression and aggressiveness.

Similarly, miR-125b-5p and miR-126-3p were consistently reported as downregulated in patients with LSCC (Hui et al. [29], Ayaz et al. [30], and Sun et al. [31]), with low levels associated with advanced clinical stages, metastasis, and poor differentiation. These miRNAs may function as tumor suppressors, and their restoration could offer therapeutic benefits. The inverse correlation between miR-126-3p and Camsap1 expression, as well as circulating tumor cell counts, suggests a mechanistic role in modulating the tumor microenvironment.

miR-27a-3p was identified as upregulated in LSCC by Ayaz et al. [30] and Shuang et al. [33], with the latter demonstrating that miR-27a-3p promotes LSCC progression via targeting Smad4 and activating the Wnt/β-catenin pathway. High miR-27a-3p levels were associated with poor prognosis, emphasizing its potential as a prognostic biomarker and therapeutic target. miR-33a-5p was reported as upregulated in patients with LSCC in the studies by Grzelczyk et al. [32] and Zhang et al. [34] and identified as a potential diagnostic biomarker with high sensitivity and specificity.

These findings underscore the complex regulatory network of miRNAs in LSCC and highlight the potential of specific miRNAs as non-invasive biomarkers for diagnosis and prognosis. The overlap in miRNA expression patterns across independent studies strengthens the validity of these miRNAs as clinically relevant biomarkers. However, variations in study designs, patient populations, and detection methods necessitate further validation in larger, more diverse cohorts.

The inclusion of studies focusing on both circulating and tissue miRNAs provides a comprehensive understanding of miRNA involvement in LSCC. While circulating miRNAs offer non-invasive diagnostic potential, tissue miRNA studies contribute to elucidating the underlying molecular mechanisms. The consistent findings between circulating and tissue miRNAs, such as miR-21-5p and miR-33a-5p, suggest that circulating miRNAs reflect tumor biology.

The majority of studies, however, focused on tissue miRNAs in regard to laryngeal cancer. For example, in the study by Gao et al. [35], the low expression of miR-145-5p combined with high levels of fascin actin-bundling protein 1 (FSCN1) correlated with poor prognosis and advanced TNM status in laryngeal squamous cell carcinoma, highlighting its role as a prognostic indicator. Similarly, Popov et al. [36] identified a distinctive proangiogenic microRNA signature, noting that miR-145-5p was significantly downregulated in advanced laryngeal carcinoma, further underscoring its impact on tumor progression and angiogenesis. Both studies reveal critical insights into the epigenetic and molecular mechanisms governing LSCC progression, presenting miR-145-5p as a key therapeutic target for modulating disease advancement and vascular dynamics.

In the study by Guo et al. [37], miR-375 was found to be a potent tumor suppressor in laryngeal squamous cell carcinoma, exhibiting significant downregulation in LSCC samples compared to adjacent non-tumor tissues. Its overexpression resulted in decreased Krüppel-like factor 4 (KLF4) levels and the marked suppression of tumor cell proliferation and invasion, and it induced apoptosis in the LSCC cell line Hep-2. Interestingly, the study also highlighted that the tumor-suppressive effect of miR-375 was more pronounced when compared to co-transfection with miR-375 and miR-206, suggesting that a single miRNA could be more effective than multiple miRNAs in certain contexts. Similarly, the study by Yu et al. [38] identified miR-375, along with miR-21 and miR-106b, as having significantly different expression levels in LSCC tissues versus adjacent normal tissues, with miR-375 being notably downregulated.

Similarly, in the study by Zhao et al. [39], miR-155 was significantly overexpressed in LSCC tissues compared to control mucosa, and this overexpression promoted the proliferation, migration, and invasion of Hep-2 cells by regulating the SOCS1-STAT3 pathway. This study also noted that miR-155 levels were particularly elevated in the more advanced T3 and T4 stages and correlated with poorer cell differentiation, suggesting its potential as a prognostic marker for LSCC progression. In a similar manner, the study by Wang et al. [40] demonstrated that both tissue and plasma levels of miR-155 were significantly higher in patients with LSCC than in controls, with tissue miR-155 showing high diagnostic accuracy (AUC of 0.933). This finding underscores the consistency of miR-155 expression across different biological matrices and its utility as a biomarker.

Other tissue miRNAs reported by multiple independent studies include miR-195. In the study by Liu et al. [41], miR-195 was found to be downregulated in laryngeal squamous cell carcinoma tissues and cell lines, and its overexpression suppressed cell proliferation, migration, and invasion by directly targeting rho-associated kinase 1 (ROCK1). This correlation of low miR-195 levels with advanced TNM stages and lymph node metastasis in patients with LSCC points to its potential role as a therapeutic target and a biomarker for aggressive disease. In a similar manner, the study by Duan et al. [42] also highlighted the role of miR-195 in LSCC, but through its interaction with circular RNA MYLK and cyclin D1. They found that circMYLK was highly expressed in LSCC and promoted tumor progression by sequestering miR-195, thereby enhancing cyclin D1 expression and facilitating cell cycle progression. These studies collectively suggest that miR-195 acts as a significant molecular brake in LSCC pathogenesis, with its downregulation linked to enhanced tumor aggressiveness, offering a potential therapeutic angle targeting the miR-195/ROCK1 and miR-195/circMYLK/cyclin D1 axes to curb LSCC progression.

Nevertheless, the standardization of miRNA detection methods and sample processing is essential for translating these findings into clinical practice. Differences in sample types (serum, plasma, tissue), extraction methods, and normalization strategies can affect miRNA quantification and comparability across studies. Establishing standardized protocols will enhance the reproducibility and reliability of miRNA-based diagnostics.

### 4.2. Limitations

Several limitations should be considered when interpreting the results of this review. Firstly, the majority of the included studies were conducted in China, with only one study from Turkey and one from Poland, potentially limiting the generalizability of the findings to other populations due to genetic and environmental differences. Secondly, there was variability in sample types (serum, plasma, tissue), detection methods (qRT-PCR, microarray, ddPCR), and study designs, which could introduce heterogeneity and affect the comparability of results. Thirdly, some studies lacked detailed patient demographic and clinical information, impacting the ability to fully assess associations between miRNA expression and clinical outcomes. The variability in sample types and collection methodologies across the included studies may affect the direct comparability of the findings. Future reviews should consider incorporating studies with more standardized methodologies to enhance the consistency and generalizability of the conclusions. Lastly, the cross-sectional nature of most studies limits the ability to establish causality between miRNA expression levels and disease progression, emphasizing the need for longitudinal studies.

## 5. Conclusions

This systematic review highlights the significant role of specific miRNAs, particularly miR-21-5p, miR-125b-5p, miR-126-3p, miR-27a-3p, and miR-33a-5p, in the diagnosis and prognosis of LSCC. The consistent findings across independent studies underscore these MiRNAs’ potential clinical utility in early detection, prognostic evaluation, and therapeutic targeting. Future research should focus on validating these miRNAs in larger, diverse populations and standardizing detection methods to facilitate their translation into clinical practice.

## Figures and Tables

**Table 1:** Study characteristics and quality assessment.

No	First Author	Year	Country	Study Design	Quality Assessment (NOS Score)
1	Gao et al. [26]	2020	China	Multicenter observational study	High (8 stars)
2	Wang et al. [27]	2014	China	Observational study	High (7 stars)
3	Wei et al. [28]	2016	China	Prospective study	High (8 stars)
4	Hui et al. [29]	2018	China	Experimental study	High (7 stars)
5	Ayaz et al. [30]	2013	Turkey	Case–control study	High (8 stars)
6	Sun et al. [31]	2014	China	Experimental study	High (7 stars)
7	Grzelczyk et al. [32]	2019	Poland	Case–control study	High (8 stars)
8	Shuang et al. [33]	2022	China	Experimental study	High (8 stars)
9	Zhang et al. [34]	2014	China	Case–control study	High (7 stars)

**Table 2:** Patient characteristics.

No	First Author	Sample Size	Mean Age (Years)	Gender (M/F)	Disease Stage	Histological Subtype	Sample Type
1	Gao et al. [26]	236 patients with LSCC	59.4 ± 10.7	223/13	T-stage I–IV; N0–N2; M0–M1	LSCC	Serum miRNA
2	Wang et al. [27]	52 patients with LSCC, 49 controls	Not specified	Not specified	Not specified	LSCC	Serum exosomal miRNA
3	Wei et al. [28]	116 patients with PLL and LSCC, 19 controls	56.9 ± 13.17	121/14	Various LSCC subgroups	LSCC	Plasma miRNA
4	Hui et al. [29]	60 patients with LSCC	Not specified	Not specified	Detailed in study	LSCC	Tissue and serum miRNA
5	Ayaz et al. [30]	20 patients with LSCC, 44 controls	Patients: 58.5 (43–76); Controls: 24.5 (18–69)	Patients: 18/2; Controls: 21/23	TNM stage I–IV	LSCC	Plasma miRNA
6	Sun et al. [31]	38 patients with LSCC	Not specified	26/12	Clinical stage I–IV	LSCC	Plasma miRNA
7	Grzelczyk et al. [32]	66 patients with LSCC, 100 controls	Not specified	Not specified	Not specified	LSCC	Serum miRNA
8	Shuang et al. [33]	62 patients with LSCC	Not specified	Not specified	T4 stage (all patients)	LSCC	Serum-derived EV miRNA
9	Zhang et al. [34]	10 patients with supraglottic carcinoma, 10 adjacent normal tissues	42–78	6/4	Not specified	Supraglottic carcinoma (LSCC subtype)	Tissue miRNA

LSCC—laryngeal squamous cell carcinoma; M/F—male/female; TNM—tumor, node, metastasis (staging system); miRNA—microRNA; EV—extracellular vesicle.

**Table 3:** miRNA expression levels and clinical associations.

No	First Author	miRNA Studied	Detection Method	Expression Levels in LSCC	Correlations
1	Gao et al. [26]	miR-21-5p, miR-10a	qRT-PCR	miR-21-5p upregulated; miR-10a downregulated	High miR-21-5p and low miR-10a associated with lower 5-year survival; correlated with TNM stages
2	Wang et al. [27]	miR-21-5p, HOTAIR	qRT-PCR	miR-21-5p and HOTAIR upregulated	Higher levels associated with advanced T classification, lymph node metastasis, and clinical stages
3	Wei et al. [28]	miR-21-5p	qRT-PCR, ddPCR	miR-21-5p significantly upregulated in PLLs and LSCC	miR-21-5p levels increased from mild dysplasia to SCC; positive correlation between tissue and plasma levels
4	Hui et al. [29]	miR-125b-5p	RT-qPCR	miR-125b-5p downregulated in LSCC tissues and serum	Low miR-125b-5p associated with advanced stage, metastasis, and poor differentiation; overexpression inhibited cell proliferation and glycolysis
5	Ayaz et al. [30]	Multiple miRNAs (e.g., miR-21-5p, miR-19a-3p, miR-125b-5p, miR-126-3p, miR-223-3p, miR-27a-3p)	qRT-PCR	miR-21-5p and miR-27a-3p upregulated; miR-125b-5p, miR-126-3p, and miR-223-3p downregulated	Altered miRNA expression profiles suggested potential as non-invasive biomarkers for LSCC
6	Sun et al. [31]	miR-126-3p	qRT-PCR	miR-126-3p downregulated in plasma	Low miR-126-3p associated with poor differentiation; inversely correlated with Camsap1 expression and CTC count
7	Grzelczyk et al. [32]	miR-31, miR-141, miR-149a, miR-182, LET-7a, miR-485-3p, miR-122, miR-33a-5p (upregulated); miR-145, miR-223-3p, miR-133a (downregulated)	Microarray, RT-qPCR	Distinct miRNA expression profile in patients with LSCC	miR-31, LET-7a, and miR-33a-5p identified as potential diagnostic biomarkers with high sensitivity and specificity
8	Shuang et al. [33]	miR-27a-3p	Microarray, RT-qPCR	miR-27a-3p upregulated in serum-derived EVs	High miR-27a-3p associated with poor prognosis; miR-27a-3p promotes LSCC progression via targeting Smad4
9	Zhang et al. [34]	miR-21-5p, miR-19a, miR-33a-5p, miR-206, miR-375	Microarray analysis	miR-21-5p, miR-19a, and miR-33a-5p upregulated; miR-206 and miR-375 downregulated in tissues	miRNA profiles could distinguish tumor from normal tissues; potential diagnostic value

miRNA—microRNA; qRT-PCR—quantitative reverse transcription polymerase chain reaction; LSCC—laryngeal squamous cell carcinoma; ddPCR—droplet digital PCR; RT-qPCR—reverse transcription quantitative polymerase chain reaction; SCC—squamous cell carcinoma; TNM stages—tumor, node, metastasis stages; EVs—extracellular vesicles; Camsap1—Calmodulin-Regulated Spectrin-Associated Protein 1; CTC—circulating tumor cell; Smad4—SMAD Family Member 4.

**Table 4:** miRNAs reported in multiple studies.

miRNA	Studies Reporting	Expression Levels in LSCC	Clinical Associations
miR-21-5p	Gao et al. [26], Wang et al. [27], Wei et al. [28], Zhang et al. [34]	Upregulated	Associated with advanced disease stages, lymph node metastasis, reduced survival
miR-125b-5p	Hui et al. [29], Ayaz et al. [30]	Downregulated	Associated with advanced stage, metastasis, poor differentiation
miR-126-3p	Ayaz et al. [30], Sun et al. [31]	Downregulated	Associated with poor differentiation; inversely correlated with Camsap1 expression and CTC count
miR-223-3p	Ayaz et al. [30], Grzelczyk et al. [32]	Downregulated	Potential diagnostic biomarker
miR-27a-3p	Ayaz et al. [30], Shuang et al. [33]	Upregulated	High levels associated with poor prognosis; promotes LSCC progression via targeting Smad4
miR-33a-5p	Grzelczyk et al. [32], Zhang et al. [34]	Upregulated	Potential diagnostic biomarker with high sensitivity and specificity

LSCC—laryngeal squamous cell carcinoma; miRNA—microRNA; CTC—circulating tumor cell.

## Data Availability

Not applicable.
